# Effects of trans-mitral flow patterns and heart rate on intraventricular pressure gradients and E/E’ in the early stage of a rat model of hypertensive cardiomyopathy

**DOI:** 10.3389/fvets.2025.1507817

**Published:** 2025-02-19

**Authors:** Miki Hirose, Danfu Ma, Kazumi Shimada, Tomohiko Yoshida, Katsuhiro Matsuura, Pitipat Kitpipatkun, Akari Hatanaka, Yanbing Zhao, Ken Takahashi, Ryou Tanaka, Lina Hamabe

**Affiliations:** ^1^Veterinary Teaching Hospital, Veterinary Medicine, Tokyo University of Agriculture and Technology, Tokyo, Japan; ^2^College of Veterinary Medicine, Nanjing Agricultural University, Nanjing, China; ^3^Department of Small Animal Medical Centre, Obihiro University of Agriculture and Veterinary Medicine, Obihiro, Japan; ^4^Department of Small Animal Clinical Sciences, University of Florida, Gainesville, FL, United States; ^5^Department of Pediatrics and Adolescent Medicine, Juntendo University Graduate School of Medicine, Tokyo, Japan; ^6^Department of Veterinary Clinical Oncology, Tokyo University of Agriculture and Technology, Tokyo, Japan

**Keywords:** diastolic function, echocardiography, heart rate, hypertensive cardiomyopathy, intraventricular pressure gradient, rat model

## Abstract

**Background:**

The mitral inflow spectral is expressed as two separate waves: early diastolic trans-mitral flow velocity (E) and late diastolic trans-mitral flow velocity (A) waves. When the heart rate (HR) increases and the diastolic time diminishes, the mitral flow pattern changes from EA-separation to EA-fusion. The E wave provides information about preload and diastolic function. Tissue Doppler imaging (TDI) and non-invasive intraventricular pressure gradient (IVPG) based on color-M-mode echocardiography are two techniques established in recent years with good repeatability in cardiac function evaluation, especially diastolic.

**Hypothesis/objective:**

We hypothesize that IVPG and E/E’ are differentially influenced by mitral inflow patterns.

**Animals:**

A total of 66 hypertensive cardiomyopathy (HTN-CM) induced by abdominal aorta coarctation and 33 sham-operated rats were divided into 6 groups according to trans-mitral flow patterns.

**Methods:**

Conventional echocardiography, TDI, and IVPG sampling were performed on rats under general anesthesia with 2.5% isoflurane at 3 weeks after the operation. After code EA-separation = 1, EA-half-separation = 2, and EA-fusion = 3, Pearson’s correlation tests were performed.

**Results:**

Both E and E’ in EA-fusion (1.04 ± 0.13 and 7.65 ± 0.84) are higher than the EA-separation pattern in all rats (0.91 ± 0.10 and 5.51 ± 0.78, *p* < 0.001). The preload change has more impact on E’ than E (0.443 vs. 0.218, p < 0.001, respectively), which leads to decreased E/E’ in EA-fusion. Total IVPG and basal IVPG positively correlated with the mitral inflow pattern (0.265 and 0.270, *p* < 0.001), while mid-to-apical IVPG was not (0.070, *p* = 0.281).

**Conclusion:**

The mitral inflow pattern positively correlates with basal IVPG, E, and E’. Mid-to-apical IVPG was independent of mitral inflow patterns, while E/E’ tended to be lower when the mitral inflow pattern changed from EA-separation to EA-fusion.

## Introduction

Hypertension was the leading risk factor for the world’s disease burden (1). Hypertensive cardiomyopathy (HTN-CM), a structural cardiac disorder resulting from hypertension, is characterized by left ventricle (LV) hypertrophy and diastolic dysfunction and is considered a silent killer with irreversible characteristics (2–4). It is crucial to diagnose HTN-CM at the early stage since morbidity and mortality synergistically increase with progressed HTN-CM (2). Although conventional echocardiography can evaluate diastolic function through mitral inflow, it is necessary to distinguish pseudo-normal patterns. The Valsalva maneuver is performed to differentiate between normal and pseudo-normal mitral flow patterns, but it is not used in small animals due to the difficulty in managing their breathing. As a result, assessing diastolic function in animals remains challenging (5, 6). The invasive catheterization test is the only method to accurately evaluate diastolic function (7). Nevertheless, the catheterization test is not useful in clinical settings because of its invasiveness, unrepeatability, time-consuming, and laborious; therefore, scientists have developed novel echocardiography technologies to monitor cardiac function non-invasively.

**Figure 1:**
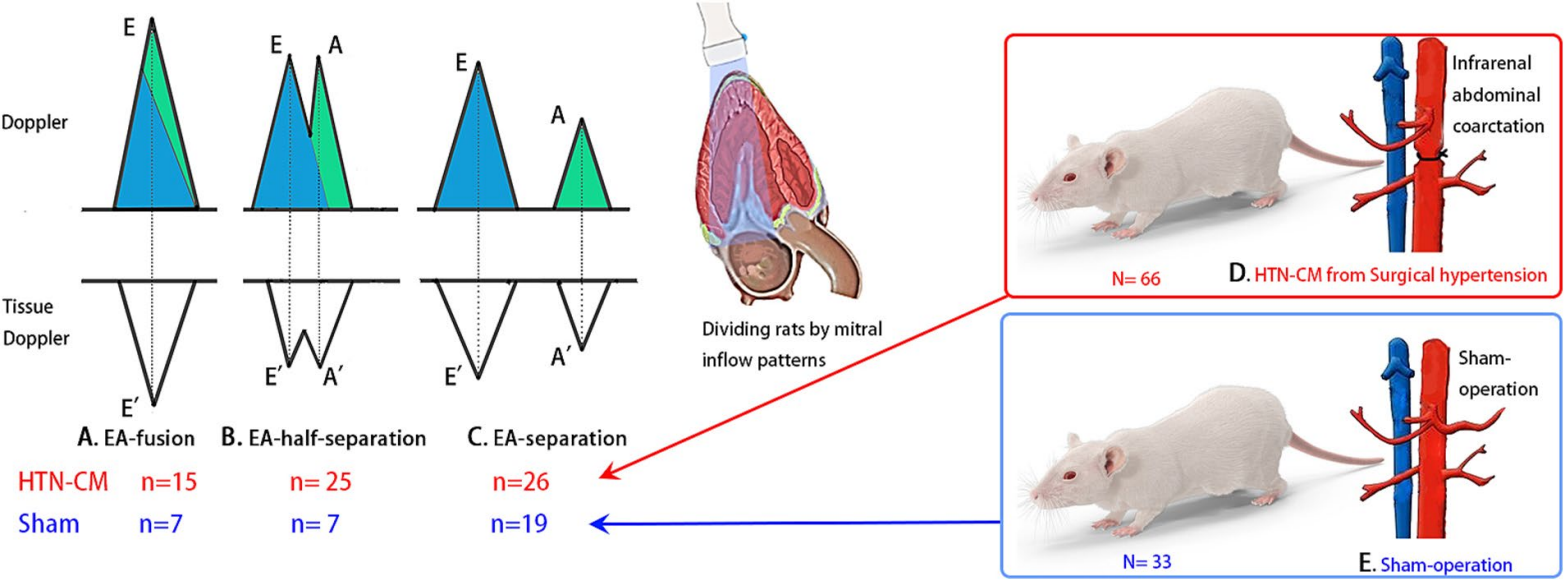
Experiment design and mitral inflow patterns: The blue color in the Doppler image indicates the volume of early diastole, and the green color represents the volume of atrial contraction. **(A)** EA-fusion pattern, indicating mixed early diastole and atrial contraction, **(B)** EA-half-separation, **(C)** EA-separation, **(D)** HTN-CM from abdominal aorta coarctation, **(E)** Sham-operation.

Elevated heart rate (HR) is a risk factor for cardiovascular morbidity and mortality in HTN-CM patients (8). In addition to the aggravation of the patient’s condition, high HR also affects the diagnosis of cardiac diseases. For instance, when the HR is elevated, diastasis is encroached or even disappears during the diastolic phase, which leads to a change of trans-mitral inflow pattern to EA-fusion (Figure 1A) or EA-half-separation (Figure 1B) from EA-separation (Figure 1C). EA-half-separation is defined as early diastole in which the atrial contraction is partially merged into the mitral inflow or myocardium movement. This pattern is regarded as the midterm status between EA-separation and EA-fusion.

Meanwhile, in the EA-fusion pattern, the early diastolic phase is completely merged with atrial contraction, such that atrial contraction is the only LV filling period (9). The early diastolic phase plays an indispensable role in LV filling because not only does it have the greatest filling volume but also the status of the early diastolic phase explains the diastolic function. Although sometimes performing echocardiography after patients calm down could avoid EA-fusion, the EA-fusion pattern is still especially common in cats and human patients (8, 10); in this scenario, conventional echocardiography, intraventricular pressure gradient (IVPG), and E/E’ are hard to judge by the veterinary clinician in EA-fusion.

Tissue Doppler imaging (TDI) is a useful tool for the quantitative assessment of systolic and diastolic function through the evaluation of the myocardium movement velocities during the cardiac cycle. However, the angle-dependent character diminishes its accuracy, and the steep learning curve for TDI data analysis limits its availability (11).

The IVPG creates a suction force that drives blood from the left atrium (LA) to the LV during early diastole. This mechanism plays a crucial role in diastole because early diastole fills approximately 80% of the ventricle volume. Therefore, IVPG is considered a diastolic indicator *in vivo*. Non-invasive IVPG measurement from color-M-mode echocardiography (CMME) using the Euler equation is well correlated with the gold standard of diastolic function parameter tau, which refers to the LV diastolic time constant (12–14). Moreover, IVPG can be used for serial assessment of diastolic function in healthy and HTN-CM animals as well as humans from infant to adult (4, 15). Thus, IVPG could easily grade HTN-CM patients using the diastolic function (4).

A previous study proved that IVPG was useful in evaluating diastolic function in hypertensive cardiomyopathy in rats (16). However, the relationship between the mitral inflow pattern and IVPG and E/E’ measurements is still unknown. Therefore, this study aimed to determine whether IVPG and E/E’ are affected by mitral inflow patterns or not and to find out the relationship between IVPG and mitral inflow patterns in HTN-CM rat models. We hypothesize that IVPG and E/E’ are differentially influenced by mitral inflow patterns.

## Materials and methods

### Animal preparation and experimental protocol

The present study was approved by the Animal Care and Use Committee of the Tokyo University of Agriculture and Technology (Approval number: 30-56), and the study was conducted under the National Guide for the Care and Use of Laboratory Animals (1994). A total of 99 healthy 10-week-old female Sprague Dawley rats (Kitayama Labes, Nagao, Japan) weighing 220–250 grams (average ± SD) were included in the study. Rats were given water and food ad libitum and kept in an air-conditioned room at 21°C under a 12-h light/dark cycle. Animals were randomly assigned to two groups: the hypertensive cardiomyopathy group (HTN-CM group, *n* = 66), created by abdominal aorta constriction, and the sham-operated control group (control group, *n* = 33).

For the HTN-CM group, an abdominal aorta coarctation operation was performed as previously described (16). The rats were anesthetized with 40 mg/kg intraperitoneal pentobarbital sodium and were placed in the dorsal position under an operating microscope (Leica M60, Wetzlar, Germany). The abdominal aorta was exposed and dissected from the caudal vena cava (above/below the renal artery) using a cotton swab. The abdominal aorta was ligated using a 3–0 silk suture with a 21-gage needle placed along the side of the aorta to avoid complete ligation (Figure 1D). The needle was removed, and after confirming blood flow had returned to the hind limb without cyanosis, the abdominal incision was closed. Rats in the control group underwent the same procedure without the ligation of the aorta (Figure 1E).

Three weeks postoperatively, blood pressure measurements, conventional echocardiography, and CMME were performed on all rats. In the HTN-CM group, hypertensive cardiomyopathy was confirmed based on the increased LV mass and elevated blood volume (3).

### Blood pressure

The non-invasive blood pressure measurement was obtained using the oscillometric method from the tail (BP monitor for rats, Muromachi, Japan), while the rats were placed in a small cage to limit their movements. Three consecutive measurements were taken, and the average of systemic systolic, diastolic, and mean arterial blood pressure was reported.

### Conventional echocardiography

To minimize the influence of anesthesia and environmental stimulation on the mitral inflow pattern and HR, rats were anesthetized with a set protocol of 2.5% isoflurane with 1 L/min oxygen supply delivered through a mask, and echocardiographic examination was carried out after the stabilization of the HR. Under general anesthesia, rats were positioned in right lateral recumbency, and an echocardiographic examination was performed in accordance with the methodology described by the European Society of Cardiology (6). The morphologic parameters, including left ventricle internal diameter in end-diastole (LVIDd) and left ventricle internal diameter in end-systole (LVIDs), were obtained from the M-mode at the right parasternal short axis view. Early diastolic trans-mitral flow velocity (E) and late diastolic trans-mitral flow velocity (A) were obtained using pulse-wave Doppler mode at the left parasternal apical four-chamber view. All measurements were taken from five consecutive heart cycles, and the average of the obtained parameters was used.

Left ventricle mass (LVM) was calculated with the formula below (6):

(1) $$\begin{array}{l} LVM=1.04\;\left[{\left(\mathrm{LVIDd}+\mathrm{IVSd}+\mathrm{LVFWd}\right)}^{3}-{\mathrm{LVIDd}}^{3}\right]\\ \times 0.8+0.6 \end{array}$$

Tissue Doppler imaging (TDI) was performed from the left parasternal apical four-chamber view. The left ventricular free wall and interventricular septal mitral annular tissue velocities were measured with a sample volume of 0.5 mm. The mitral annular tissue velocity profile, which includes early diastolic tissue velocity (E’) and late diastolic tissue velocity (A’) at the interventricular septum (IVS) and left ventricular posterior wall (LVFW), was obtained.

E/E’ was calculated by the below formula:

(2) E/E′=EE′atIVS+EE′atLVFW2

LV end-diastolic pressure (LVEDP) and LV diastolic pressure at the onset of the a-wave (Pre-A LVDP) were calculated by the linear regression equation from invasive catheterization and E/E’ (17).

(3) $$\mathrm{LVEDP}=17.1+0.19*{\frac{E}{E}}^{’}$$

(4) $$Pre-A\;\mathrm{LVDP}=6.97+0.3*{\frac{E}{E}}^{’}$$

### Color-M-mode echocardiography for assessment of IVPG

**Figure 2:**
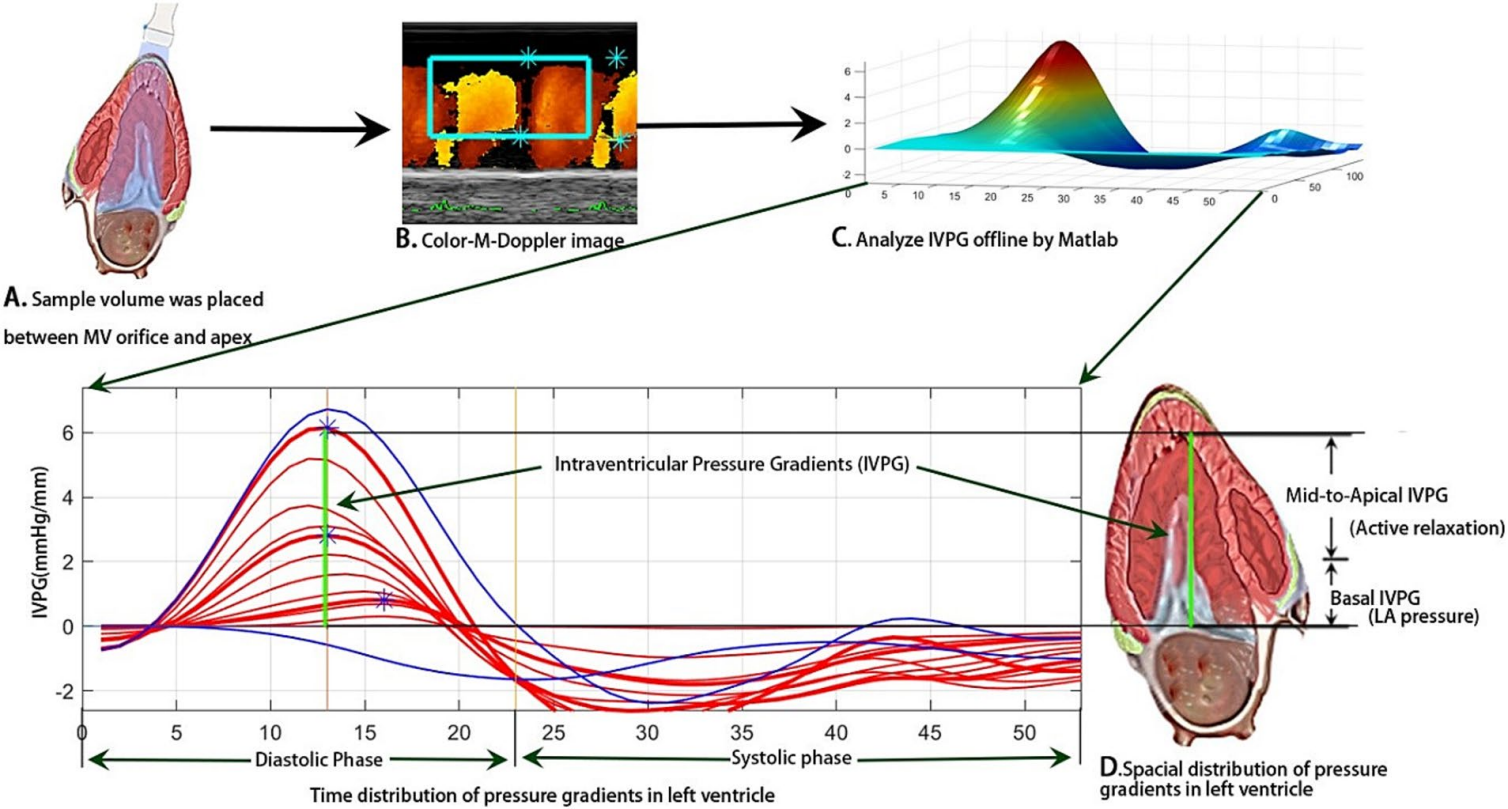
Schematic illustration of IVPG measurement through image analysis of photographs obtained from CMME in left apical four-chamber view using MATLAB software.

CMME was performed from the left parasternal apical four-chamber view under the left recumbence gesture. The sample volume was parallel to the mitral inflow in the apical view (Figure 2A) using an ultrasound system (Hitachi Aloka Medical ProSound Premier 75CV, Japan), which supports the CMME (Figure 2B) with a 12-MHz probe. Prior machine setting was conducted for the proper tracing of the CMME, which includes a sweep speed of 300 mm/s, and a color baseline shift to −64 to increase the Nyquist limit. All images were analyzed with the Euler equation using MATLAB (Figure 2C, The MathWorks, Natick, MA) as previously described (15, 18), and the intraventricular pressure differences (IVPDs) were obtained as follows:

$$\left(\partial \;P\right)/\left(\partial s\right)=-\rho \left(\left(\partial v\right)/\left(\partial \;t\right)+v\;\left(\partial v\right)/\left(\partial \;s\right)\right)$$

where *∂* is the change in element followed, P is the pressure, ρ is the constant blood density (1,060 kg/m^3^), *v* is the velocity, s is the position along with the color M-mode line, and t is the time. The IVPG values were derived from the IVPDs according to the following formula (16, 19):

$$\mathrm{IVPG}\;\left(\mathrm{mmHg}/\mathrm{cm}\right)=\mathrm{IVPD}/LV\;\mathrm{length}\text{.}$$

The total IVPG was divided into two segments based on one-third segments of LV length: The smaller segment near the mitral valve is the basal IVPG, and the mid-to-apical IVPG segment is the other two-thirds near the apical IVPG (Figure 2D). Basal IVPG was proven to be correlated with LA pressure, and mid-to-apical IVPG represents active relaxation of the LV (13).

### Animal classification

Based on the mitral inflow pattern, the Sham-operated and HTN-CM rats were further divided into six groups (three groups in each category) as follows: EA-fusion (*n* = 7, 15), EA-half-separation (*n* = 7, 25), and EA-separation (*n* = 19, 26), respectively, for Sham and HTN-CM (Figures 1A–C).

### Statistical analysis

All data were measured at least five times and are expressed as mean ± SD. The group (Sham vs. HTN-CM) and mitral inflow patterns (EA-fusion, EA-half-separation, and EA-separation) were considered as two variables used for comparison by two-way ANOVA, and Turkey’s test was used for the *post-hoc* test. A *p*-value of < 0.05 was considered statistically significant. SPSS (IBM, Armonk, New York) was used to analyze the relationship between cardiac parameters and novel echocardiographic parameters.

### Correlation tests

A prior conversion of the subjective variables, including mitral inflow patterns, into numerical variables was performed before conducting the correlation analysis. For instance, the mitral inflow pattern is coded as 1 = EA-separation, 2 = EA-half-separation, and 3 = EA-fusion. Then, the IVPG, E/E’, E, and E’ were taken as variables to perform correlation tests with HR and mitral inflow patterns.

## Results

### Conventional echocardiography

Conventional echocardiography parameters are displayed in Table 1 and Supplementary Table S1. The HTN-CM group showed elevated blood pressure (160.79 vs. 90.81, *p* < 0.001) and LVM compared with the Sham group (0.86 vs. 0.64, *p* < 0.001), respectively. The combination of these measurements confirmed the existence of HTN-CM. In addition, no difference was detected in the ejection fraction, which indicates a stable systolic function in HTN-CM rats. As expected, HR and E in EA-fusion rats were significantly higher than in EA-separation rats in both groups.

**Table 1:** Comparison of conventional echocardiographic parameters in the Sham and HTN-CM groups regarding the pattern of the mitral inflow.

Group	Sham group			HTN-CM group		
Patterns parameters	EA-fusion (*n* = 7)	EA-half-sep. (*n* = 7)	EA-sep. (*n* = 19)	EA-fusion (*n* = 15)	EA-half-sep. (*n* = 25)	EA-sep. (*n* = 26)
LVM (g)	0.62 ± 0.13^c^	0.62 ± 0.11^c^	0.65 ± 0.13^c^	0.83 ± 0.23^ab^	0.86 ± 0.31^a^	0.87 ± 0.35^a^
EF (%)	75.13 ± 5.12^a^	71.93 ± 6.8^a^	70.37 ± 10.63^a^	70.21 ± 9.68^a^	70.9 ± 8.33^a^	65.33 ± 6.14^a^
E (m/S)	1.01 ± 0.13^abc^	0.96 ± 0.11^bcd^	0.90 ± 0.10^d^	1.06 ± 0.13^a^	1.02 ± 0.16^ab^	0.93 ± 0.09^cd^
A (m/S)	/	/	0.63 ± 0.16	/	/	0.64 ± 0.11
HR (bpm)	359.72 ± 42.3^a^	315.77 ± 54.10^b^	304.14 ± 43.97^b^	350.32 ± 40.39^a^	348.48 ± 36.11^a^	306.22 ± 39.68^b^
E/A ratio	/	/	1.57 ± 0.09	/	/	1.49 ± 0.04
E’ (cm/S)	7.74 ± 0.84^a^	5.67 ± 0.71^bc^	5.51 ± 0.81 ^c^	7.59 ± 1.13^a^	6.22 ± 1.03^b^	5.40 ± 0.76^c^
E/E’	13.21 ± 2.13^b^	17.27 ± 2.52^a^	16.68 ± 2.64 ^a^	14.47 ± 2.93^b^	16.91 ± 2.78^a^	17.79 ± 2.85^a^
LVEDP (mmHg)	19.61 ± 0.41^b^	20.38 ± 0.48^a^	20.27 ± 0.50 ^a^	19.61 ± 0.41^b^	20.31 ± 0.53^a^	20.48 ± 0.54^a^
Pre-A LVDP (mmHg)	10.93 ± 0.64^b^	12.15 ± 0.76^a^	11.97 ± 0.79 ^a^	11.31 ± 0.88^b^	12.04 ± 0.83^a^	12.31 ± 0.85^a^
SAP (mmHg)	95.05 ± 9.02^c^	89.28 ± 11.4^c^	89.79 ± 13.9^c^	169.26 ± 18.69^ab^	166.38 ± 13.42^a^	150.76 ± 19.64^b^

Echocardiographic measurements in sham and hypertensive cardiomyopathy (HTN-CM) rats after 3 weeks. Each group was classified according to the mitral inflow pattern. The group (Sham vs. HTN-CM) and mitral inflow patterns (EA-fusion, EA-half-separation, and EA-separation) were considered as two variables which were used for comparison by two-way ANOVA. The Turkey test was used for post-hoc comparison. The letters are fitted for comparing means between groups, and different letters in the same raw indicate significant differences (*p* < 0.05). sep., separation; LVM, left ventricle mass; E, the velocity of early mitral inflow; Peak A, the velocity of late mitral inflow; HR, heart rate; E’, peak velocity of early diastolic mitral annular motion as determined by pulsed-wave Doppler; E/E’, ratio of E to E’; LVEDP, left ventricle end-diastolic pressure; Pre-A LVDP, left ventricle diastolic pressure at the onset of the A-wave; SAP, systolic arterial pressure.

### Tissue Doppler imaging

In sham and HTN-CM rats, elevated myocardium movement and mitral inflow velocity indicate hyperdynamic states in EA-fusion pattern rats (Table 1). E/E’ in the HTN-CM rats was higher than in Sham rats in EA-fusion and EA-separation patterns and higher than 15, although not significant. E/E’ was lower in EA-fusion rats compared with EA-half-separation and EA-separation groups, respectively, in sham (13.21 vs. 16.68, *p* < 0.001) and HTN-CM rats (14.47 vs. 17.79, *p* < 0.001). As a result, the LVEDP and pre-A LVDP of EA-fusion groups in the Sham and HTN-CM rats were significantly lower than the LVEDP and pre-A LVDP of the EA-half-separation groups and EA-separation groups in the Sham and HTN-CM rats (*p* < 0.001 and *p* < 0.001).

### IVPG measurements

**Figure 3:**
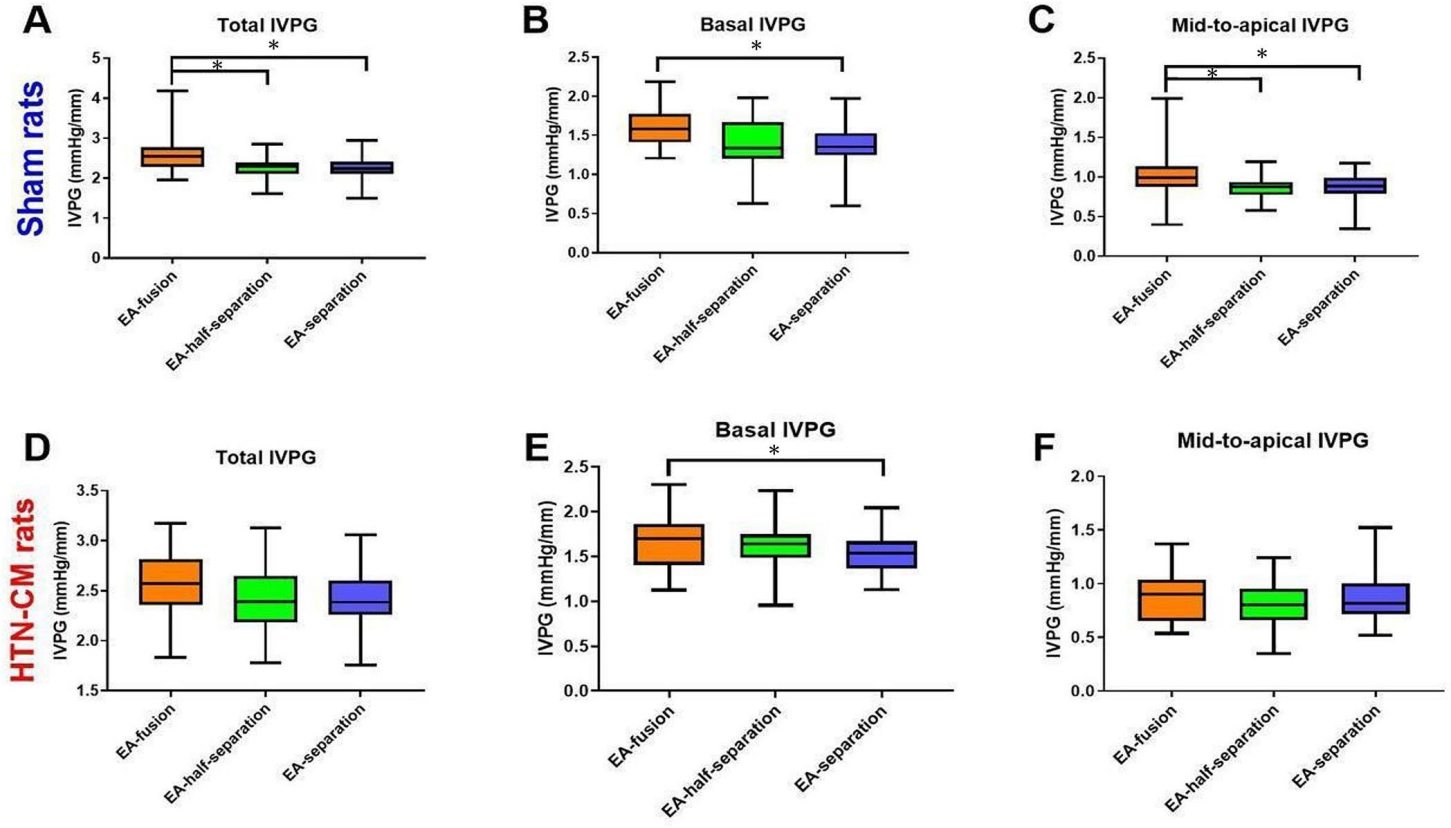
Comparison of IVPG data according to the mitral inflow pattern (EA-fusion, EA-half-separation, and EA-separation) in the Sham **(A–C)** and HTN-CM **(D–F)** groups. *p < 0.05.

The IVPG data are summarized in Figure 3. In the sham group, the total IVPG, basal IVPG, and mid-to-apical IVPG were significantly higher in EA-fusion rats than in EA-separation rats (2.61, 1.60, 1.01 vs. 2.26, 1.38, 0.88, *p* < 0.001, *p* = 0.008, 0.015), respectively. Furthermore, EA-fusion rats were significantly higher than EA-half-separation rats in the sham group in terms of the total IVPG and mid-to-apical IVPG (2.61 and 1.01 in EA-fusion vs. 2.27 and 0.86 in EA-half-separation, *p* = 0.005 and 0.024). Meanwhile, in HTN-CM rats (Figures 3D–F), only basal IVPG showed a significant difference between EA-fusion and EA-separation rats (1.68 vs. 1.54, *p* = 0.027).

### Pearson’s correlation test of the novel echocardiography

Table 2 shows the correlation results between E/E’, IVPG, and other cardiac parameters. HR showed a highly significant positive correlation with total IVPG (*p* < 0.001), basal IVPG (*p* < 0.001), mid-to-apical IVPG (*p* = 0.003), E (*p* < 0.001), and E’ (*p* < 0.001) average; meanwhile, HR revealed a highly significant negative correlation with E/E’ (*p* < 0.001). Because the mitral inflow pattern is transferred from string variables into numerical variables, the correlation (r) is a relative value rather than an absolute value. In this regard, the mitral inflow patterns showed a significant positive correlation with total IVPG (*p* < 0.001), basal IVPG (*p* < 0.001), E (*p* < 0.001), and E’ (*p* < 0.001). Meanwhile, E/E’ showed a significant negative correlation with the mitral inflow pattern (*p* < 0.001). In other words, E/E’ tends to be lower when the mitral inflow pattern changes from EA-separation to EA-fusion. The HR also correlates with the mitral inflow pattern.

**Table 2:** Pearson’s correlations test of echocardiography.

Variables	HR		Mitral inflow pattern	
	*r*	*p*	*r*	*p*
Total IVPG	0.412^**^	<0.001	0.265^**^	<0.001
Basal IVPG	0.358^**^	<0.001	0.270^**^	<0.001
Mid-to-apical IVPG	0.188^**^	0.003	0.070	0.281
E/E’	−0.291^**^	<0.001	−0.381^**^	<0.001
E	0.218^**^	<0.001	0.389^**^	<0.001
E’	0.443^**^	<0.001	0.665^**^	<0.001
Mitral inflow pattern	0.434^**^	<0.001	/	/

The relationship between E/E’ and IVPG data and mitral inflow pattern as well as groups. **p < 0.05. Total IVPG, the mix of basal and mid-to-apical intraventricular pressure gradients; basal IVPG, the basal intraventricular pressure gradients; mid-to-apical IVPG, the mid-plus apical intraventricular pressure gradients; HR, heart rate; E/é, E to mitral annulus velocity ratio; E, the velocity of early mitral inflow; E’ average, average early diastolic mitral annulus velocity.

## Discussion

In clinical settings, physicians generally perform echocardiography when the HR is calm and stable to avoid EA wave fusion caused by high HR. Previous studies proved that E and A were related to HR and atrioventricular conduction delay, and E and A were proved influenced by ventricular loading and diastolic property (20, 21). However, the effect of mitral inflow patterns on novel echocardiography techniques such as IVPG has never been explored before. Thus, when the EA-fusion happens, physicians and scientists fail to gain valuable information from novel echocardiography techniques such as IVPG and E/E’. So, we designed this study to reveal whether IVPG and E/E’ are affected by mitral inflow patterns or not. We hypothesized that mitral inflow patterns would affect IVPG and E/E’ differentially. Our results showed, for the first time, that IVPG was weakly influenced by mitral inflow patterns, while mitral inflow patterns affect E/E’.

In this experiment, elevated blood pressure supports hypertension, and combined with the elevated LVM, HTN-CM is recognized in the HTN-CM group (3). In hypertension patients, the baseline resting HR is higher than 85 bpm, leading to a shrunk value on echocardiography because of the changed mitral inflow pattern (8, 22). In the current study, we chose the surgical hypertension model to mimic early-stage HTN-CM patients in this study (23). The heart rate of awake rats is approximately 300–400 bpm, but the heart rate of rats anesthetized with isoflurane was an average of 326 ± 46 (219–421). Because HTN-CM rats responded differently to anesthesia, instead of intentionally controlling the depth of anesthesia to obtain a stable HR, we performed echocardiography at the same isoflurane dose to confirm that the effect of anesthesia was the same in all rats.

The different mitral inflow patterns had different IVPG values, as shown in Figure 3. In EA-fusion rats, the pressure built up by accumulated pulmonary venous blood in the atrium combined with the left atrial contraction resulted in promoted LV inflow speed, volume, and LV myocardium movement (24). The total and basal IVPG elevation explained the promoted myocardium movement in EA-fusion rats. The basal IVPG is well connected to LA pressure. Similarly, elevated LA pressure and volume in EA-fusion rats led to promoted basal IVPG (13). In the present study, mid-to-apical IVPG was significantly increased in EA-fusion rats in the sham group. The development of compensated hypertrophy leads to non-significantly increased mid-to-apical IVPG in HTN-CM rats. The mitral inflow pattern is not correlated with mid-to-apical IVPG because mid-to-apical IVPG illustrates active relaxation, which stays at the same level when the mitral inflow pattern changes.

E/E’ was well correlated with Pre-A LVDP and LVEDP (17). The LVEDP is the same thing as Pre-A LVDP in EA-fusion rats because the E wave is merged with the A wave in EA-fusion patterns. However, based on the theoretical calculation, the LVEDP was approximately twice the Pre-A LVDP in EA-fusion rats, which showed the challenge of using E/E’ to speculate LVEDP and Pre-A LVDP in EA-fusion rats.

The result of Pearson’s correlation test indicates that the mitral inflow pattern correlates with HR. When HR increases, the mitral inflow changes from EA-separation to EA-fusion, and the total IVPG, basal IVPG, E, and E’ average also increases.

However, the E/E’ is negatively associated with HR because the E’ is elevated more than the E velocity. The EA-fusion pattern owns the lowest preload among the three patterns because it owns only one inflow, while the EA-separation pattern has two mitral inflows. Similarly, preload affects E’ velocity more than E velocity (17), and we proved that the mitral inflow patterns correlate more with E’ than E (*r* = 0.443 vs. 0.218, Table 2). Because E/E’ was calculated by E velocity and E’, the changed mitral inflow patterns led to the uncertainty of E/E’. This explains why mitral inflow patterns affected E/E’. This study helps physicians and scientists better understand conventional and novel echocardiography techniques such as E, E/E’, and IVPG when mitral inflow is fused into one flow.

### Study limitations

In the present study, only female rats were included in this study. Although both genders develop hypertension, males have a higher incidence and severity of hypertension compared with peer females until they are 60 or over (1, 25). However, the physiological differences, including growing speed, in young rats may interfere with the homogeneous HTN-CM model (26).

## Conclusion

Elevated HR leads to encroached diastasis, which eventually leads to EA-fusion. We proved that HR is associated with the mitral inflow pattern. Both E and E’ in EA-fusion are higher than the EA-separation pattern. The preload change has more impact on E’ than E, which leads to decreased E/E’ in EA-fusion. HR positively correlates with basal IVPG, E, and E’, while mid-to-apical IVPG is independent of mitral inflow patterns. It is not necessary to control HR to obtain IVPG because the impact of HR on IVPG is weak.

## Data Availability

The original contributions presented in the study are included in the article/Supplementary material, further inquiries can be directed to the corresponding authors.
