# Supplementary material for: Effects of trans-mitral flow patterns and heart rate on intraventricular pressure gradients and E/E’ in the early stage of a rat model of hypertensive cardiomyopathy
Source: Front Vet Sci. 2025 Feb 19;12:1507817. doi: 10.3389/fvets.2025.1507817 (PMC11879942; doi:10.3389/fvets.2025.1507817)
Supplement: Supplementary file 1 [file Table_1.DOCX]

Table S1. Conventional echocardiography before and after operation

| Group | Sham rats before opearation (n=33) | HTN-CM rats before opearation (n=66) | Sham rats (n=33) | HTN-CM rats (n=66) |
| --- | --- | --- | --- | --- |
| IVSd (mm) | 1.17±0.14^#^ | 1.21±1.06^#^ | 1.19±0.15^#^ | 1.65±0.35^*ǂ&^ |
| LVIDd (mm) | 7.19±0.54 | 7.16±1.19 | 7.36±0.69 | 7.07±0.75 |
| LVPWd (mm) | 1.48±0.3^#^ | 1.51±0.33^#^ | 1.48±0.31^#^ | 1.88±0.66^*ǂ&^ |
| IVSs (mm) | 1.99±0.33^#^ | 2.08±0.53^#^ | 1.96±0.34^#^ | 2.45±0.41^*ǂ&^ |
| LVIDs (mm) | 4.16±0.54^#^ | 4.17±0.58^#^ | 4.22±0.64^#^ | 4.07±0.63 |
| LVPWs (mm) | 2.27±0.33^#^ | 2.55±6.83^#^ | 2.31±0.37^#^ | 2.68±0.41^*ǂ&^ |
| FS (%) | 42.14±4.96 | 41.75±5.22 | 42.71±6.73 | 41.7±12.6 |
| E (cm/S) | 95.8±12.1 | 93.87±12.62 | 93.41±11.98 | 99.41±14.3 |
| A (cm/S) | 62.87±13.28 | 60.68±15.85 | 62.49±16.57 | 63.95±10.92 |
| E’ septum (cm/S) | 5.67±1.35 | 5.65±1.22 | 5.73±1.26 | 5.76±1.32 |
| E’ free wall (cm/S) | 6.36±1.46 | 6.25±1.39 | 6.31±1.29 | 6.63±1.49 |
| A’ septum (cm/S) | 5.41±0.65 | 5.82±1.02 | 5.67±1.02 | 5.95±0.97 |
| A’ free wall (cm/S) | 6.56±1.07 | 6.24±1.31 | 6.27±1.15 | 6.57±1.29 |
| SAP (mmHg) | 95.05±9.02 | 93.39±11.97^#^ | 90.81±12.6^#^ | 114.87±14.12^*ǂ&^ |
| DAP (mmHg) | 56.43±7.46 | 60.21±8.32^#^ | 53.54±8.53^#^ | 82.65±9.85^*ǂ&^ |
| MAP (mmHg) | 68.61±9.89 | 71.26±10.12^#^ | 65.65±4.51^#^ | 91.64±6.71^*ǂ&^ |

Echocardiographic measurements according to the mitral inflow patterns in sham and HTN-CM rats after 3 weeks of operation using one-way ANOVA. * indicates significant difference with Sham rats, # indicates significant difference with HTN-CM rats, ǂ indicates significant difference with sham rats before the operation, & indicates significant difference with HTN-CM rats before operation (*p*<0.05). IVSd, interventricular septum diastolic diameter; LVIDd, left ventricular internal diastolic diameter; LVPWd, left ventricular posterior wall diastolic diameter; IVSs, interventricular septum systolic diameter; LVIDs, left ventricular internal systolic diameter; LVPWs, left ventricular posterior wall systolic diameter; a’ septum, late septum movement; a’ free wall, late free wall movement; A’ average, average late atrial movement; SAP, systolic arterial pressure; DAP, diastolic arterial pressure; MAP, mean arterial pressure.
